# Investigation of the Efficiency of Mask Wearing, Contact Tracing, and Case Isolation during the COVID-19 Outbreak

**DOI:** 10.3390/jcm10132761

**Published:** 2021-06-23

**Authors:** Tatiana Filonets, Maxim Solovchuk, Wayne Gao, Tony Wen-Hann Sheu

**Affiliations:** 1 Department of Engineering Science and Ocean Engineering, National Taiwan University, Taipei 10617, Taiwan; tfilonets@gmail.com (T.F.); twhsheu@ntu.edu.tw (T.W.-H.S.); 2 Institute of Biomedical Engineering and Nanomedicine, National Health Research Institutes, No. 35, Keyan Road, Zhunan 35053, Taiwan; 3 College of Public Health, Taipei Medical University, Taipei 11031, Taiwan; waynegao@tmu.edu.tw

**Keywords:** COVID-19, non-pharmaceutical interventions, case isolation, contact tracing, mask wearing, outbreak control

## Abstract

Case isolation and contact tracing are two essential parts of control measures to prevent the spread of COVID-19, however, additional interventions, such as mask wearing, are required. Taiwan successfully contained local COVID-19 transmission after the initial imported cases in the country in early 2020 after applying the above-mentioned interventions. In order to explain the containment of the disease spread in Taiwan and understand the efficiency of different non-pharmaceutical interventions, a mathematical model has been developed. A stochastic model was implemented in order to estimate the effectiveness of mask wearing together with case isolation and contact tracing. We investigated different approaches towards mask usage, estimated the effect of the interventions on the basic reproduction number (R_0_), and simulated the possibility of controlling the outbreak. With the assumption that non-medical and medical masks have 20% and 50% efficiency, respectively, case isolation works on 100%, 70% of all people wear medical masks, and R_0_ = 2.5, there is almost 80% probability of outbreak control with 60% contact tracing, whereas for non-medical masks the highest probability is only about 20%. With a large proportion of infectiousness before the onset of symptoms (40%) and the presence of asymptomatic cases, the investigated interventions (isolation of cases, contact tracing, and mask wearing by all people), implemented on a high level, can help to control the disease spread. Superspreading events have also been included in our model in order to estimate their impact on the outbreak and to understand how restrictions on gathering and social distancing can help to control the outbreak. The obtained quantitative results are in agreement with the empirical COVID-19 data in Taiwan.

## 1. Introduction

Currently, the world is gripped by a new pandemic called COVID-19. As of 13 January 2021, according to the WHO [1], the number of confirmed cases is 90,335,008, and the number of deaths is 1,954,336. Before global vaccination, countries should conduct non-pharmaceutical interventions to control and further prevent the spread of the virus.

One of the main non-pharmaceutical measures includes case isolation and contact tracing. The experience of the struggle against another respiratory disease, SARS [2], demonstrated that the use of these two interventions could prevent the spread of infection. However, studies have shown that isolation and tracing are effective if infectiousness is only possible after the occurrence of symptoms, as in the case of SARS. If the infection appears before the start of symptoms (pre-symptomatically), as in the case of COVID-19, these measures begin to lose their effectiveness along with an increase of the transmission probability before the onset of symptoms. One work showed that, on average, 44% of infected people (95% confidence interval, 25–69%) infect others before symptoms appear [3]. This result proves that additional non-pharmaceutical measures are needed to prevent COVID-19, such as social distancing, wearing masks, and following personal hygiene rules, including frequent hand washing and temperature checking in public places.

All of these non-pharmaceutical measures are recommended by the WHO [4,5], while the attitude towards masks and their role in blocking the spread of coronavirus has changed significantly over the period from April to June 2020. At the beginning of April, WHO advised wearing a medical mask if you had respiratory symptoms and should perform hand hygiene after disposing of the mask [4]. Then in early June, there was a separate guide about wearing masks in which masks were recommended to be worn by healthy people as well [5], to reduce the potential risk of infection. This guide also indicates the possible transmission of coronavirus from people who have not yet developed symptoms or who have never had them (asymptomatic cases). In this regard, WHO advises that people, even without any respiratory symptoms, should also wear masks to reduce the potential risk of infection [5].

The virus transmission from asymptomatic cases also plays a very important role in epidemic spread, but an estimation of the proportion and infectivity of these cases is still unclear. Some recent papers assessed that asymptomatic people constituted approximately 40–50% of all confirmed COVID-19 cases [6,7,8]. Another study estimated this fraction to be approximately 15% [9]. Asymptomatic cases can spread the disease for a much longer time period (more than 14 days) than symptomatic cases [6,10]. The infectiousness of asymptomatic individuals is still uncertain, but a meta-analysis and an investigation of antibody responses to COVID-19 infection demonstrated that asymptomatic individuals might be less contagious than symptomatic cases [8,10]. The existence of pre-symptomatic and asymptomatic transmission makes the COVID-19 epidemic more challenging to control and prevent than SARS. One of the most recent studies highlights the importance of worldwide testing [6]. We propose that another way to achieve control of this outbreak is mask wearing.

At the beginning of 2021, the vaccination program started in some countries [11]. Vaccination can lead to a decrease of the virus spread and relaxation of the non-pharmaceutical interventions (NPIs). However, a recent study estimated the effect of vaccination and weakening of control measures, demonstrated that early relaxation of NPIs, before sufficient immunity has been established, would provoke a large wave of infection [12]. Thus, investigations of different NPIs and their efficiency are still necessary even during the vaccination period.

During the first wave of COVID-19 in January–March 2020, Taiwan successfully contained local COVID-19 transmission without implementing unprecedented and costly interventions such as social distancing measures and the lockdown of regions and cities. During January–March 2020 Taiwanese government and CDC immediately started implementation of strict non-pharmaceutical interventions to control the virus spreading (case isolation, tracing of contacts, provision of medical mask to population and hard control for mask wearing in public places) [13]. The aim of the current study is to analyse these interventions implemented by the government in Taiwan and investigate their efficiency. The effectiveness of non-pharmaceutical interventions such as public mask-wearing for eradicating community COVID-19 transmission in the absence of city lockdowns and the restriction of individual movement presents an important lesson for further outbreaks.

Increasing attention to the role of masks in the fight against the spread of the disease is based on recent studies [14,15,16,17], which explored how the virus spreads around infected people when they breathe, speak, or cough, and tested the ability of the mask to block virus spread and to provide protection from infected individuals. In one study [14], the authors demonstrate that simple surgical masks worn by individuals with upper respiratory tract infections could dramatically reduce the viral concentration in their exhaled air. Physicists from Bauhaus University, Weimar, recorded a video in which they showed how far air particles can travel when someone breathes and coughs with or without a mask [18]. This video simply shows that masks limit how far away respiratory particles can travel. In another study [15], the authors investigated the spread of droplets during a speech using a laser device and showed how masks can reduce this spread.

It is worth mentioning that people can also use homemade face masks. This may happen when there is a lack of surgical masks or the government tries to reserve, in the first place, masks for healthcare facilities. The effectiveness of homemade masks depends on the type of cloth and the number of layers [19,20]. Some recent studies showed that non-medical masks can be much less effective than medical masks [21,22,23].

These studies provided motivation for a possible assessment of the effect of mask wearing on controlling the COVID-19 outbreak. For this purpose, we used a stochastic model and evaluated the impact of non-pharmaceutical interventions such as case isolation, contact tracing, and the wearing of masks [24]. Recently it was found that there are so-called superspreaders (individuals producing a larger number of cases than a reproduction number) [25]. These superspreading events have been included in our model in order to estimate their impact on the outbreak and to understand how restriction in gathering can help to control the outbreak. By varying the scenarios and probabilities of mask wearing, we demonstrated that the wide-spread use of masks by the general public can be considered as one of the important control measures in addition to basic preventative interventions: case isolation and contact tracing. Our predicted results are in agreement with the COVID-19 situation in Taiwan, where application of the above-mentioned interventions helped to almost eliminate the local virus spread during 2020 year [13], and allows to suppress the exponential growth of the new wave started in May 2021 [26].

## 2. Methods

### 2.1. Stochastic Transmission Model

**Figure 1:**
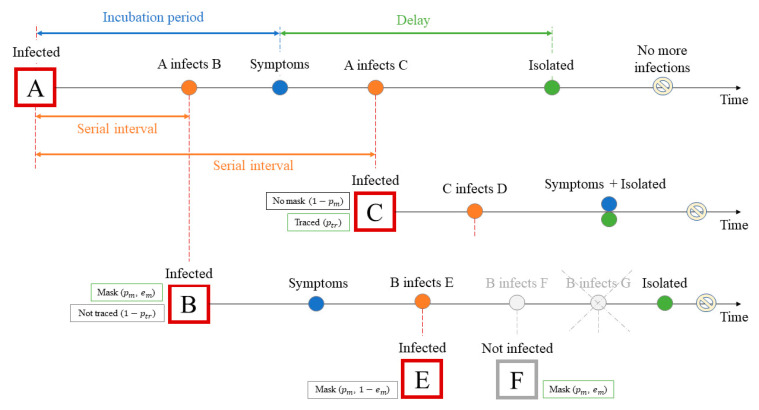
Scenario example for the simulated branching process. Initially, there is one infected person A who does not wear a mask and who was infected at time t=0. A infected B before the symptoms onset, who then infects C after symptom onset and is isolated with a certain delay. In its turn, the new case C infects several people before it is isolated. However, since the case C is traced, its time of isolation comes earlier than it would have been without tracing (for this example, the isolation time coincides with the appearance of symptoms). Case B, unlike case C, is not traced, but the person wears a mask, which reduces the number of secondary cases that it can produce: instead of three new cases, B infects only two (E and F cases). In this example, we suppose the mask can be used for source control and for the protection of healthy persons from infection with some efficiency $e_{m}$. Therefore, the person F does not get infected because of the mask, while for person E, the mask does not work, and E becomes infected. Thus, case B, instead of the possible three potential new cases, produces only one new case.

We used a stochastic model, that is, a branching process, in order to analyse the effects of different interventions on the epidemic spread: case isolation, contact tracing, and mask wearing. The model is based on the branching process used by Hellewell et al. [24]. Figure 1 shows an example of one of the scenarios for the progress of this branching process. In this model, the number of potential new cases produced by each individual is drawn from a negative binomial distribution (Supplementary Figure S1a) with a mean value equal to the reproduction number $R_{0}$, dispersion parameter equal to k = 0.1 [27], and variance R_0_(1 + R_0_/k). This distribution has a long right-hand tail and is highly overdispersed (for R_0_ = 2.5 standard deviation is 8), in which the smaller the k, the longer the tail and, therefore, the greater the heterogeneity [28,29]. The heterogeneity allows to assume a presence of superspreading events (SSEs) when one person can infect a much larger number of people than R_0_ [25,30]. However, the probability of appearing SSEs is not so high, for example, for R_0_ = 2.5 probability of infection more than 10 people by one individual is around 0.7% (Supplementary Figure S1a).

Similar analyses using branching process models have already been used to analyse the Wuhan outbreak to find plausible ranges for the reproduction number and for the initial exposure event size [24,31,32]. Our analysis complements these works as follows—we included masks and different options of wearing them to estimate the impact of mask wearing. Additionally, asymptomatic transmission has been added and recent epidemiological parameters of asymptomatic and pre-symptomatic transmission have been used [3,8,10].

### 2.2. Threshold Value for $R_{0}$ and Outbreak Control

The basic reproduction number $R_{0}$, the average number of new infections generated by each infected individual, is directly related to the type and intensity of interventions necessary to control an epidemic [33]. High values of $R_{0}$ mean it is easy to transmit the disease and low values indicate it is difficult to transmit. There are three options for assigning the values of the reproduction number. It can be less than one, which means that the number of new cases will decrease over time, and eventually the outbreak will be eliminated. It can be equal to one, which means the number of cases will be stable over time. In addition, it can be greater than one, which means that the outbreak is self-sustaining unless effective control interventions are implemented. Therefore, the objective of public health efforts is to achieve $R_{0}$ < 1 as soon as possible [33].

To assess the impact of non-pharmaceutical measures (case isolation, contact tracing, mask wearing), we evaluated the effective reproduction number ($R_{\mathrm{eff}}$) and outbreak control for each simulation [24]. $R_{\mathrm{eff}}$ is defined as the average number of people that one infected person can infect when the specified control measures are in effect. An outbreak is considered to be controlled if the number of new cases did not exceed 5000 during the first three months (12 weeks), and no new cases were detected during the following four months (between 12 and 16 weeks). Note that by new cases we mean only symptomatic cases, since asymptomatic cases are practically not reported. In addition, they can be affected by isolation or contact tracing only if their “parents” (from whom they got infection) are symptomatic cases.

### 2.3. Time Data for the Transmission Model

For each potentially new infected person, the following time moments are set: a time of infection exposure, a time of symptoms onset, and an isolation time (for a symptomatic person). All these times are taken from their own distribution functions (Supplementary Figure S1b–d). Case isolation is assumed to work with 100% efficiency, which means that after isolation, infected people cannot infect anyone. In addition to the case isolation, another intervention is included in this model—contact tracing. This allows, with a certain probability $p_{tr}$, finding and isolating newly infected individuals. The isolation time for traced cases is defined as follows:

(1) $$\min\left(t_{s}+d,\;\max\left(t_{s},\;t_{iso}^{infector}\right)\right),$$

where $t_{s}$ is the symptoms onset time, $t_{iso}^{infector}$ is the infector’s isolation time, and d is the delay between $t_{s}$ and the isolation time without contact tracing.

### 2.4. Mask Wearing

Considering the changing attitudes about the effectiveness of mask use, there are three different scenarios of mask wearing:

(1) Masks reduce the spread of infection and keep germs contained (source control) [14,15,16,17,18], so only sick people wear the mask with a certain probability [4];
(2) Masks reduce the spread of infection and keep germs contained (source control) [14,15,16,17,18], but since studies show that the COVID-19 virus can be spread by a person who does not have symptoms [3], masks are worn by all people with a certain probability;
(3) Masks both reduce the spread of infection from an infected person (source control) and prevents the spread of infection to a healthy person (protection of the wearer) [5,17]. Therefore, in this scenario, both infected and susceptible persons wear masks with a certain probability.

We suppose that the mask has some effectiveness $e_{m}$. It is also assumed that people can wear masks with a certain probability $p_{m}$.

As mentioned above, the mask reduces the number of new potential cases, i.e., the $R_{0}$ for the infected individual who wears the mask decreases to $R_{0}^{mask}$:

(2) $$R_{0}^{mask}=R_{0}\left(1-e_{m}\right).$$

We also tried to consider the quality of wearing a mask (whether a person uses a mask correctly or not, whether they often take it off when talking or locate in public places, etc.). For this, we use the Bernoulli distribution with a success probability equal to the mask efficiency $e_{m}$. Thus, if the infected person wears the mask correctly, then he will have his $R_{0}$ counted according to Equation (2), whereas if he wears it incorrectly, his *R*_0_ will not change. The same applies to susceptible people: the mask will not protect them against infection if it is worn in the wrong way.

The effectiveness of the medical mask was taken to be 50%, although different authors showed that the effectiveness of the surgical mask can range from 40–50% to 90–100% [14,34]. The efficacy of the non-medical masks depends on their material and design [35], and it can vary between 2% and 38% [22]. We took 20% efficiency for a homemade mask but also, we investigated the influence of other mask efficiency values (from 10% to 90%).

### 2.5. SARS-CoV-2 Infection Parameters

According to one study [3], the estimated proportion of pre-symptomatic transmission is 44% (95% CI, 25–69%). In our model, we assumed a 40% fraction of transmission before symptoms onset. At the present moment, there are quite a few estimates of the reproduction number $R_{0}$ for different countries [31,32,36,37,38]; we took three different values of $R_{0}$: 1.5, 2.5, and 3.5.

The CDC (Centres for Disease Control and Prevention) estimated the infectiousness of asymptomatic individuals relative to symptomatic individuals based on studies of viral shedding dynamics [39]; this value is equal to 75%. Therefore, we assumed that the reproduction number for asymptomatic cases ($R_{a}$) is equal to 75% of the $R_{0}$. Using the recent estimation of the infection shedding period for asymptomatic cases [10], we suppose that on average this period is equal to 19 days. Another study assessed that symptomatic people can make asymptomatic cases with 15% probability, whereas for asymptomatic persons this probability is equal to 50% [8].

Due to people gathering and some social events, SSEs can occur more frequently in real life. The distribution of secondary cases by negative binomial distribution considers the relatively small probability of SSEs, which can be seen as some kind of a restriction on a gathering of a large number of people. Wong and Collins estimated distribution for COVID-19 SSEs, which is consistent with Fréchet distribution with tail parameter α = 1.7 [25], and we explored this scenario by combining negative binomial and Fréchet distributions. In this scenario, the probability of infecting a larger than R_0_ number of people slightly increases (Supplementary Figure S1a).

We wrote the C++ program for the calculations of the results and validated it by comparing these with the results of Hellewell et al. [24]. The code is available in GitHub at https://github.com/FilkinaGramota/Stochastic-Model (accessed on 22 June 2021).

All values of the main parameters used in this work are shown in Table 1.

## 3. Results

Since this model involves a stochastic process, to obtain the results, we ran 1000 simulations for different combinations of the parameters presented in Table 1.

### 3.1. Effective Reproduction Number

**Figure 2:**
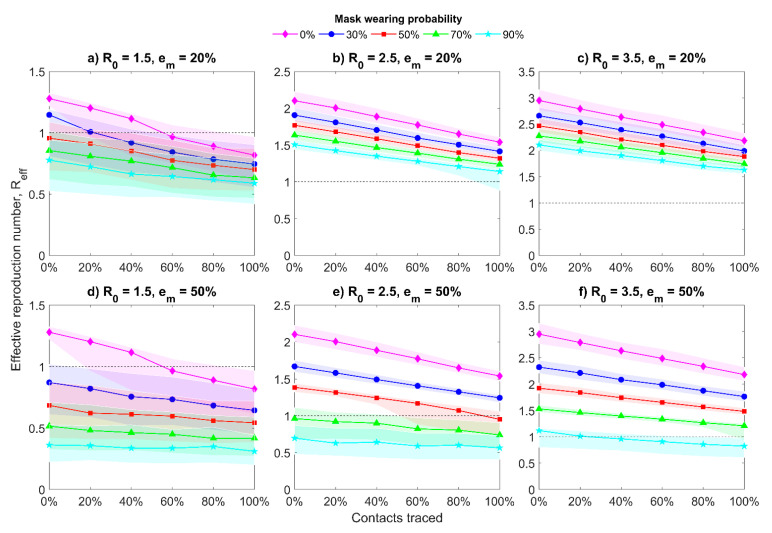
Median of effective reproduction number with interquartile range. Twenty initial symptomatic cases, 20 initial asymptomatic cases, all people wear masks, and the mask hinders spreading and protects from getting infected with different mask efficiency $e_{m}$, $R_{a}$ = 0.75·$R_{0}$, 4 days of mean delay, and 40% transmission before symptoms. Top row graphics (**a**–**c**) represent results for homemade masks, bottom row graphics (**d**–**f**) represent results for medical masks.

Figure 2 demonstrates the changes in the values of effective reproduction number *R*_eff_ with interquartile range (IQR) in the presence of asymptomatic cases and when all people wear masks with different efficiency $e_{m}$. Under the phrase “all people” we consider that infected and uninfected, pre-symptomatic, symptomatic, and asymptomatic cases, as they can all wear masks but with a different probability. For initial value $R_{0}$ = 2.5 (Figure 2b,e), we notice that with increasing percentages of traced contacts the median value decreases relatively slow, especially when homemade masks are used. Seventy percent probability of medical mask ($e_{m}$ = 50%) wearing by population can bring $R_{\mathrm{eff}}$ to one without taking into consideration the contact tracing. As it was expected, the situation is much better for the smaller initial value $R_{0}$ = 1.5 (Figure 2a,d): even without mask usage, the $R_{\mathrm{eff}}$ declines below one for more than 60% of the contacts traced (for medical and non-medical mask). While for the larger initial value $R_{0}$ = 3.5, even a 70% probability of mask wearing together with 100% traced contacts can provide the $R_{\mathrm{eff}}$ value only close to one (Figure 2c,f). It means that for $R_{0}$ = 3.5 we should use a very high probability of mask wearing (for example, 90%). For all considered cases, the reproduction number for asymptomatic cases $R_{a}$ = 0.75·$R_{0}$.

**Figure 3:**
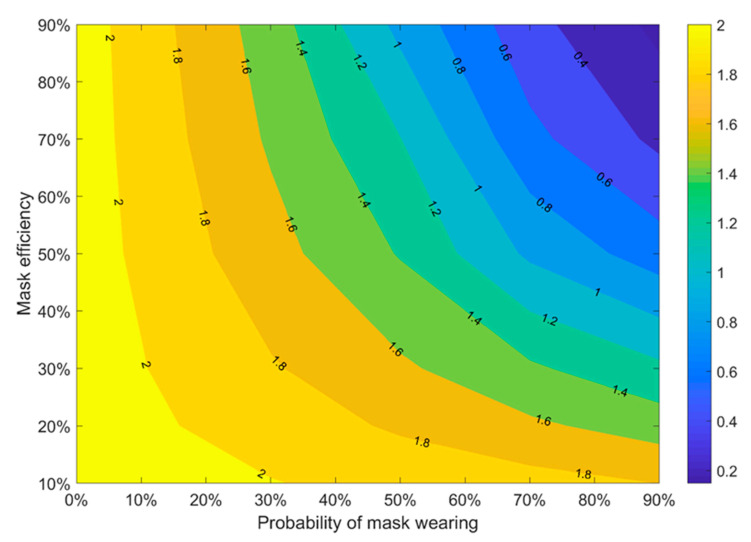
Median of effective reproduction number with the only presence of 100% case isolation (without using contact tracing and people gathering restrictions). Twenty initial symptomatic cases, 20 initial asymptomatic cases, initial $R_{0}$ = 2.5, $R_{a}$ = 1.875, all people wear masks, and the mask hinders spreading and protects from getting infected with different mask efficiency, 4 days of mean delay, 40% transmission before symptoms.

To show the impact of mask wearing on the reproduction number, we investigated the dependency of $R_{\mathrm{eff}}$ on the mask properties (efficiency and wearing probability). The results are represented in Figure 3. Here we suppose that besides mask wearing, only one another intervention is applied: the isolation of new cases. It means that contact tracing and restriction in the gathering are not taken into account in Figure 3. We can see that the best results (the smallest $R_{\mathrm{eff}}$) can be reached when both mask efficiency and wearing probability are higher than 80%. At the same time, we can see that $R_{\mathrm{eff}}$ < 1 when the probability of mask wearing is greater than 70% and mask efficiency is at least 50%, which is close to the efficiency of medical masks. These results can be useful during the vaccination period when some control measures may be relaxed, which may invoke a new COVID-19 wave [12]: maintaining a high-quality case isolation, together with a high proportion of people wearing medical masks, may reduce the breeding rate to a value of less than one.

### 3.2. Outbreak Control

**Figure 4:**
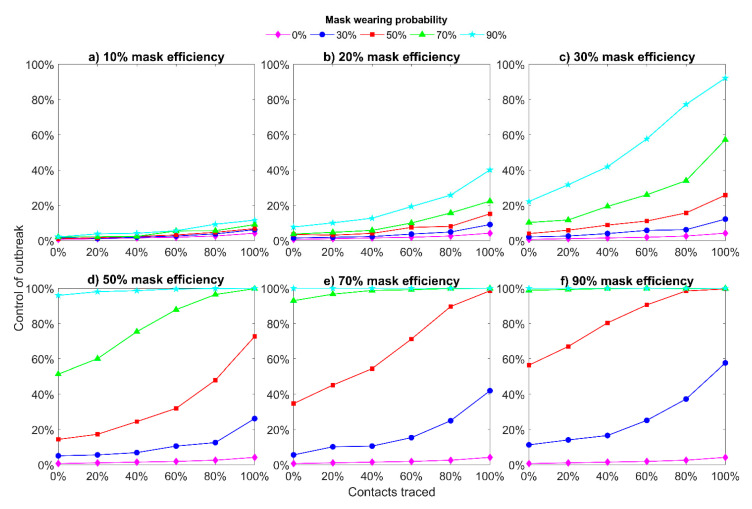
Outbreak control for different mask efficiency. All people wear masks and the mask hinders spreading and protects from getting infection with different efficiency. 20 initial symptomatic cases, 20 initial asymptomatic cases, initial $R_{0}$ = 2.5, $R_{a}$ = 1.875, 4 days of mean delay, 40% transmission before symptoms.

Figure 4 shows the probability of achieving the outbreak control for different mask efficiency and mask wearing probability. We can see that for a small value of mask efficiency (<30%, Figure 4a–c), this intervention (mask wearing) cannot help to control an outbreak even when a large proportion of contacts are traced. Only perfect contact tracing (100%) together with 90% of people wearing masks can help to achieve around 90% probability of outbreak control. However, it is almost impossible to implement it in reality. For higher values of mask efficiency (Figure 4d–f), we can achieve a high probability of outbreak control (70% or more) with a lower percentage of traced contacts. If we consider that the mask prevents spreading and protects from getting infection with 90% efficiency (Figure 4f), then the outbreak is totally controlled when only case isolation is used (0% of contacts are traced) and people wear masks with more than 70% probability.

### 3.3. Superspreading Events

**Figure 5:**
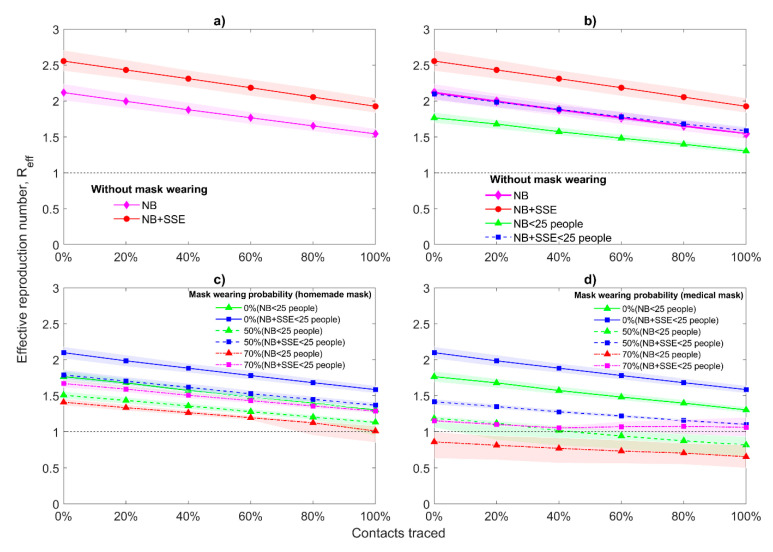
Median of effective reproduction number with interquartile range and superspreading events. $R_{0}$ = 2.5, $R_{a}$ = 1.875, 4 days of mean delay, 40% transmission before symptoms, initial number of symptomatic and asymptomatic cases is the same and equal to 20, all people wear masks, and the mask hinders spreading and protects from getting infected with different mask efficiency $e_{m}$. Distributions used for secondary cases: negative binomial (NB), negative binomial with additional superspreading events (NB+SSE), negative binomial with restriction on maximum cases (NB < 25 people), negative binomial with additional superspreading events and restriction on maximum cases (NB + SSE < 25 people): (**a**) without mask wearing and SSEs; (**b**) without mask wearing but with SSEs; (**c**) $e_{m}$ = 20% (homemade mask); (**d**) $e_{m}$ = 50% (medical mask).

As was mentioned in Methods, we have also investigated the scenario when there are additional SSEs by coupling negative binomial and Fréchet distribution (Supplementary Figure S1). Consideration of additional SSEs in the model makes decrease of $R_{\mathrm{eff}}$ more challenging and weakens the effects of other interventions: case isolation, tracing, and mask wearing (Figure 5). Clusters of transmission from one person to a potentially large group are particularly important for maintaining the virus’s spread. Therefore, restrictions in gatherings may lower the average reproduction number value and the tail of the negative binomial distribution substantially. Restrictions in gathering can be taken into account in the model by limiting the maximum amount of people (25 people in our case) that one individual can infect. Without mask wearing, joint application of restriction in gathering, case isolation, and contact tracing can decrease the value of $R_{\mathrm{eff}}$ only up to 1.5 (Figure 5b). If we also take into consideration that 50% of population wears homemade masks, then $R_{\mathrm{eff}}$ can be close to one but only with 100% of contact tracing (Figure 5c). However, if 50% of people will wear medical masks, then for 60% contact tracing we can find that $R_{\mathrm{eff}}$ < 1 in the absence of additional SSEs (Figure 5d). Increasing the probability of mask wearing to 70%, for non-medical masks, $R_{\mathrm{eff}}$ still cannot overcome the threshold value 1 (Figure 5c). For medical masks and without additional SSEs, $R_{\mathrm{eff}}$ is less than one without any contact tracing whereas in the presence of supplemental SSEs $R_{\mathrm{eff}}$ fluctuates near one but does not cross this. Despite this fact, the results with restrictions are much better than without, where even with 90% probability of homemade mask wearing and 100% contact tracing $R_{\mathrm{eff}}$ cannot reach the threshold value (Figure 2).

Moreover, if we make restrictions stricter, for example, one person cannot infect more than 15 people, then $R_{\mathrm{eff}}$ can be less than one when 70% of people wear homemade masks and more than 60% of contacts are traced (Supplementary Figure S2). These results show the importance of each intervention and the powerful effect of their combinations when all control measures perform on a high level. Moreover, the combination of these control measures demonstrates its effectiveness even in situations when people can use only non-medical masks.

### 3.4. Comparison with Official Taiwanese Data

**Figure 6:**
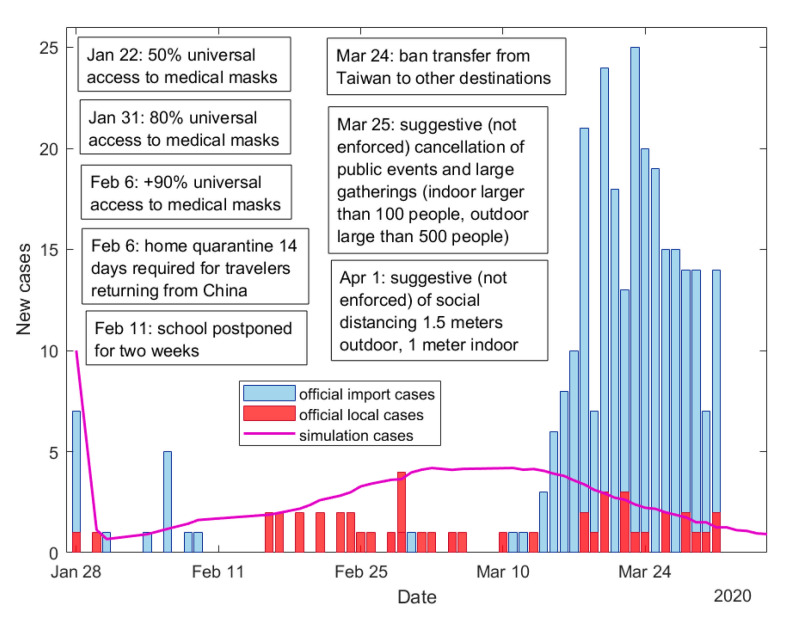
Comparison of official data in Taiwan during January–March 2020 with simulated results and implemented interventions in Taiwan during this period [13]. $R_{0}$ = 2.5, $R_{a}$ = 1.875, 4 days of mean delay, 40% transmission before symptoms, initial number of symptomatic and asymptomatic cases is the same and equal to 5, 80% of people wear masks and the mask hinders spreading and protects from getting infected with mask efficiency $e_{m}$ = 50%, 60% of contacts are traced.

At the beginning of the COVID-19 epidemic in January 2020, Taiwan immediately implemented non-pharmaceutical control measures (Figure 6) such as distribution of medical masks to the population, mandatory mask wearing in public places, contact tracing, case isolation, and provide high-quality two weeks quarantine in separate places with doctor checking and following monitoring of a person for one week [13,42]. According to the estimation of the cases, which were detected by contact tracing program using mobile messages and applications, 61.5% of cases were revealed in Taiwan because traced contacts [43]. We compared results of our model and empirical data of Taiwan, when the first wave in Taiwan had only just started. During January–March 2020 in Taiwan there were more imported cases than local (Figure 6). Figure 6 shows good agreement of the model results with official data of local cases. For modelling, we used parameter values that were estimated for Taiwan for this period: probability of medical mask wearing is 80% [13] and proportion of traced contacts is 60% [43]. Results from Figure 6 prove that early implementation of non-pharmaceutical interventions in a quite high level can help to contain the local virus spread.

## 4. Discussion

We have investigated three different types of interventions (case isolation, contact tracing, and mask wearing) that together can provide help to control a new COVID-19 outbreak. We found that in some scenarios, case isolation together with contact tracing was not enough to control the spread of the disease. The feasibility of controlling the outbreak by case isolation and mask wearing depends on the fraction of pre-symptomatic transmissions, on the fraction of asymptomatic cases, on their infectiousness, and on the time delay between symptoms appearance, and isolation initiation: the smaller these parameters are, the higher the effectiveness of these interventions. Since masks efficiency can be varied from 10% to 90% depending on the quality of a mask [14,22,34], the type of masks and the proportion of the population wearing masks are also very important factors. We also investigated the effect of the restrictions on people gathering, which is also one of the important interventions used to fight the COVID-19 outbreak.

The wearing of medical masks plays an essential role in the assistance of outbreak control. A high probability of medical mask wearing (70% or more) allows for control of the situation by more than 80% likelihood with a moderate level of contact tracing. Using non-medical masks with the 70–90% wearing probability can provide outbreak control at about 20–40% level with very good contact tracing. These results, from one point of view, are in agreement with recommendations that highly effective contact tracing will be necessary to control outbreaks in other countries [24,44]. From another point of view, they also demonstrated the effectiveness of mask wearing [14], and when a high percentage of the population wears masks (70% or above) a chance to disease control is increased. It is worth mentioning that as for any other interventions, contact tracing with mask wearing requires certain expenditures from the authorities [45]. However, the implementation of contact tracing program, such as 80–100% of people will be traced, is too hard and less possible than encouraging people to wear medical masks everywhere. If there is very large number of infected cases and it is not possible to implement contact tracing at high level, strict restrictions on people gathering (and social distancing) can be introduced to reduce the infected number. After that, these restrictions can be eased when a certain level of contact tracing is reached.

Our study shows that mask wearing (especially medical) can be used as one of the additional control measures to the basic interventions (case isolation and contact tracing) to reduce COVID-19 spread and prevent infection over a longer period. However, to achieve a high probability of outbreak control all three interventions are required to be implemented together and with very good quality. Taiwan and South Korea demonstrated that these control measures can really help in the control of the outbreak [45,46]. For example, Taiwan’s Central Epidemic Command Center implemented rapid case finding and hospital isolation, tracing, and testing the close contacts of infected individuals very early on [45], was able to contain the first wave of COVID-19 and prevent the second wave. The Taiwanese industry was also able to open several new mask production lines, with production capacity increasing more than four times in two months (from the end of January to the end of March of 2020 year) which allowed to supply all of the population with surgical masks [42]. As a result, already on 6 February 2020, universal access to medical masks in Taiwan reached 90% [13]. It can be seen that our predicted probabilities of the outbreak control (Figure 4c) are consistent with the real local outbreak control in Taiwan, where a local spread of the virus has been maintained at a low level without country lockdown [13].

The recent spike of transmissions in Taiwan reached more than 200 on May 16, 2021. In the context of this article, this wave can be explained by the weakening of contact tracking and the fact that people are wearing masks less and are also not wearing them correctly. The daily confirmed cases have been relatively stable between 200 and 300 cases and have remained contained in Taiwan after June 6, 2021 (Supplementary Figure S4) without witnessing an expected exponential growth in cases and hospitalization [47]. This further proves that the control measures have effectively kept the basic reproduction number reasonably around the value of one. This can be explained by rapid tightening of contact tracing and wearing of masks measures [47].

In Figure 4 and Figure 5, it can be seen that when using medical masks (efficiency of 50%), the probability of achieving epidemic control is much higher with a 70% probability of wearing masks than with a 50% probability. Thus, the weakening of at least one intervention can lead to a new outbreak. Therefore, it can be concluded that the state and people should be very attentive to each other and observe basic control measures (masks, contact tracing, rapid isolation of cases) until the global epidemic subsides to zero.

The results with additional SSEs and with the restriction in gathering (on the maximum number of people that can be infected by one individual) can show not only a very important role of combination of all basics control measures (case isolation, contact tracing, mask wearing, restriction on a gathering of a large number of people) but also demonstrate that removal of different restrictions should be performed very carefully and after careful consideration. Our results demonstrate that a slight increase in the probability of SSEs appearing weakens the effects of other interventions. Whereas the addition of restrictions on people gathering improves the exposure of other control measures (especially when a strict restriction is used).

The model and the obtained results demonstrate that generally wearing medical masks (or another type of mask with high efficiency) by a large proportion of the population can be useful for the struggle against the COVID-19 epidemic. However, the effect of masks depends not only on the fabric quality and number of layers but also on us: how often and how correctly we wear masks, and how frequently we change them.

Our study has some limitations. We introduced only one version of the definition of the controlled outbreak (during 3 months with no more than 5000 cases, following the approach presented in the study of Hellewell et al. [24]) but did not investigate the behaviour of an outbreak for a longer period. In our study, we considered a restriction on people gathering by using a very simple approach—as the restriction on the maximum number of people infected by one person. However, in reality, the SSEs and their restrictions are more complex since many different places can be a source of SSEs and the corresponding restrictions can be applied in different ways [48].

In addition, we want to mention that besides investigating the current manuscript interventions, there are simple and very useful precautions which WHO, ECDC, CDC urge us to follow, such as keeping a distance of about 6 feet from other people, ventilation of rooms [49], washing hands, and to clean and disinfect frequently touched surfaces daily [19,50,51]. These measures are not even primarily for our own health; they are for the health of our family, neighbours, and other people.

## Figures and Tables

**Table 1:** Parameter values used in the model.

	Value		Reference
Fixed			
Initial symptomatic cases	5; 20; 40; 50; 100		Tested
Initial asymptomatic cases	0; 20; 50; 100		Tested
Reproduction number $R_{0}$	1.5; 2.5; 3.5		[24,32,38]
Dispersion parameter for negative binomial distribution	0.1		[27]
Tail parameter for Fréchet distribution	1.7		[25]
Isolation efficiency	100%		[24]
Proportion of transmission before symptoms onset	40%		[3]
Proportion of asymptomatic cases (produced from symptomatic)	15%		[8]
Proportion of asymptomatic cases (produced from asymptomatic)	50%		[8]
Infectivity of asymptomatic cases $R_{a}$	0.75·$R_{0}$		[39]
Percentage of contacts traced	0%; 20%; 40%; 60%; 80%; 100%		[24]
Mask efficiency $e_{m}$	10%; 20%; 30%; 50%; 70%; 90%		[14,22,34]
Mask wearing probability	0%; 30%; 50%; 70%; 90%		Tested
**Sampled**	**Mean**	**Standard deviation**	
Incubation period	5.75	2.63	[40]
Serial interval	Incubation period	2	[24]
Delay between symptoms onset and isolation time	3.83	2.38	[41]
The infection shedding period for asymptomatic cases	19	8.15	[10]

## Data Availability

The authors confirm that the data supporting the findings of this study are available within the article and its supplementary material. All code for the model is available at GitHub: https://github.com/FilkinaGramota/Stochastic-Model (accessed on 22 June 2021).

## Supplementary Materials

The following are available online at https://www.mdpi.com/article/10.3390/jcm10132761/s1.
