# Supplementary material for: Investigation of the Efficiency of Mask Wearing, Contact Tracing, and Case Isolation during the COVID-19 Outbreak
Source: J Clin Med. 2021 Jun 23;10(13):2761. doi: 10.3390/jcm10132761 (PMC8269102; doi:10.3390/jcm10132761)
Supplement: Supplementary file 1 [file jcm-10-02761-s001.zip › jcm-1245862-SI.pdf]

Supplementary material

1. Probability distribution functions used in the model

The number of potential new cases produced by each individual is drawn from a negative binomial (NB) distribution with a mean value equal to the reproduction number R_0 and dispersion parameter equal to 0.1 (median 0.1; 95% CrI: 0.05–0.2 for R_0 = 2.5) [1]. One of the advantages of this model is that it takes into account the overdispersed data, which have a variance $R_0(1+R_0/k)$ larger than the mean value [2]. This fact can be interpreted as considering the population heterogeneity.

During the pandemic, there are superspreading events (SSEs) which are characterized by certain persons infecting a greater number of people relative to R_0. The recent study estimated the distribution of SSEs for COVID-19 [3]. We tried to combine NB distribution with additional SSEs (Figure S1a). We can see that these extra SSEs decrease the probability of infect 0–2 people whereas the probability of infections 6, 8, or more than 10 persons slightly increases.

To determine the delay between the onset of symptoms and isolation, the Weibull distribution is used [4]. The incubation period by which the onset of symptoms is determined also has a Weibull distribution [5].

To determine when an infection is received, a serial interval is defined that obeys a skewed normal distribution. To determine the fraction of the pre-symptomatic cases, the moment of infection must depend on the time of the infector's symptoms onset, i.e., the serial interval and the incubation period must be related to each other. Therefore, for each new case, its own function of the skewed normal distribution is constructed with a mean equal to the incubation period and a standard deviation equal to 2 [6]. The skew parameter represents the proportion of transmission before symptoms onset.

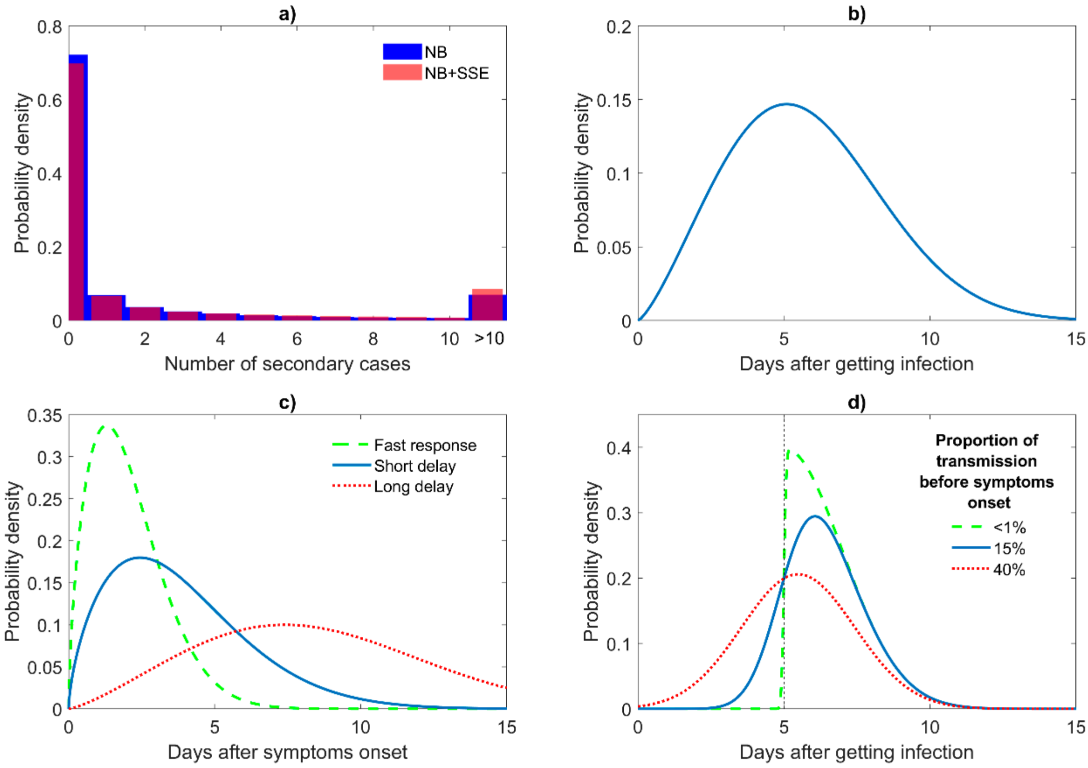

Supplementary Figure S1. Probability distribution functions. **a)** negative binomial distribution for potential new cases with (NB+SSE) and without (NB) additional superspreading events; **b)** distribution for the incubation period; **c)** distribution for the delay between symptoms onset and isolation; **d)** distribution of the serial interval with different proportions of transmission before symptoms onset (the dashed line indicates the moment of symptoms onset)

2. Effect of additional superspreading events and restriction on gathering people on the reproduction number

Figure S2 shows changes in the values of effective reproduction number R_{eff} (the average number of people that one infected person can infect when the control measures are in effect) with interquartile range (IQR) in the presence of additional superspreading events (SSEs) and with the restriction on gathering (with the introduced maximum number of people that can be infected by one individual) [3]. We can see that for relatively strict restriction on the maximum number of people that can be infected by one infected person (no more than 15) and the high proportion of contact tracing (more than 60%), the wearing of homemade masks by 70% of the population can lead R_{eff} under the threshold value without additional SSEs (Figure S2c). With extra SSEs, a higher proportion of contact tracing (more than 80%) is required in order to control the outbreak for homemade masks. For medical masks, R_{eff} decreases faster, and quite good results can be observed for a small percentage of contact tracing (Figure S2d).

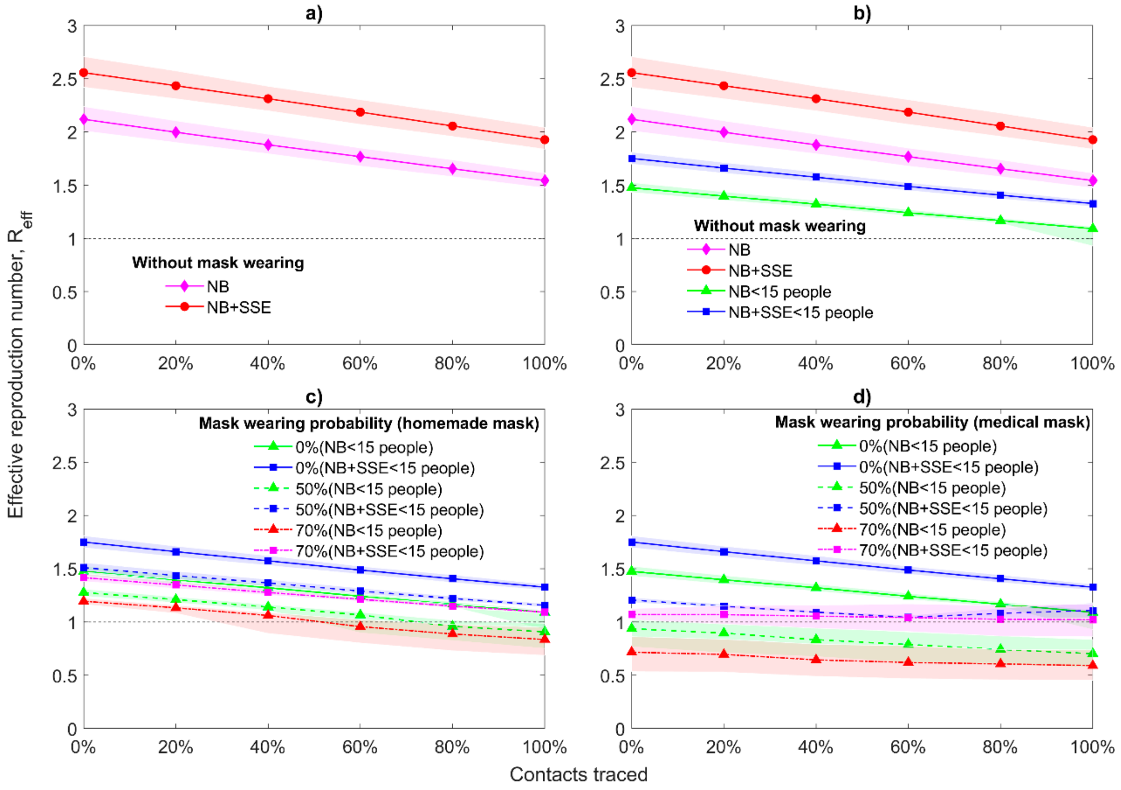

Supplementary Figure S2. Median of effective reproduction number with IQR, additional superspreading events and restriction on 15 maximum cases that can be produced by one person. 20 initial symptomatic cases, 20 initial asymptomatic cases, all people wear masks and the mask hinders spreading and protects from getting infection with mask efficiency 20% (homemade mask) and 50% (medical mask), initial $R_0 = 2.5$, $R_a = 1.875$, 4 days of mean delay, 40% transmission before symptoms. Distributions used for secondary cases: negative binomial (NB), negative binomial with additional superspreading events (NB+SSE), negative binomial with restriction on maximum cases (NB<15 people), negative binomial with additional superspreading events and restriction on maximum cases (NB+SSE<15 people).

3. Outbreak control for different number of initial cases

Increasing the initial number of infected cases can make the control of the outbreak more difficult. For a larger number of initial (symptomatic and asymptomatic) cases, a 30% likelihood of mask wearing does not have a strong impact and cannot help to control the outbreak, even when 100% of contacts are traced (Figure S3). At the same time, for non-medical masks and high 70% mask wearing probability, the outbreak control can be achieved with less than 5% possibility and with around 12% for an improbable situation when 90% of people wear masks. When 70% of the population wear surgical masks, even for a high number of initial cases, we can achieve a fairly high probability of a controlled outbreak (70% or more) but only together with excellent case isolation and a medium fraction of the traced contacts.

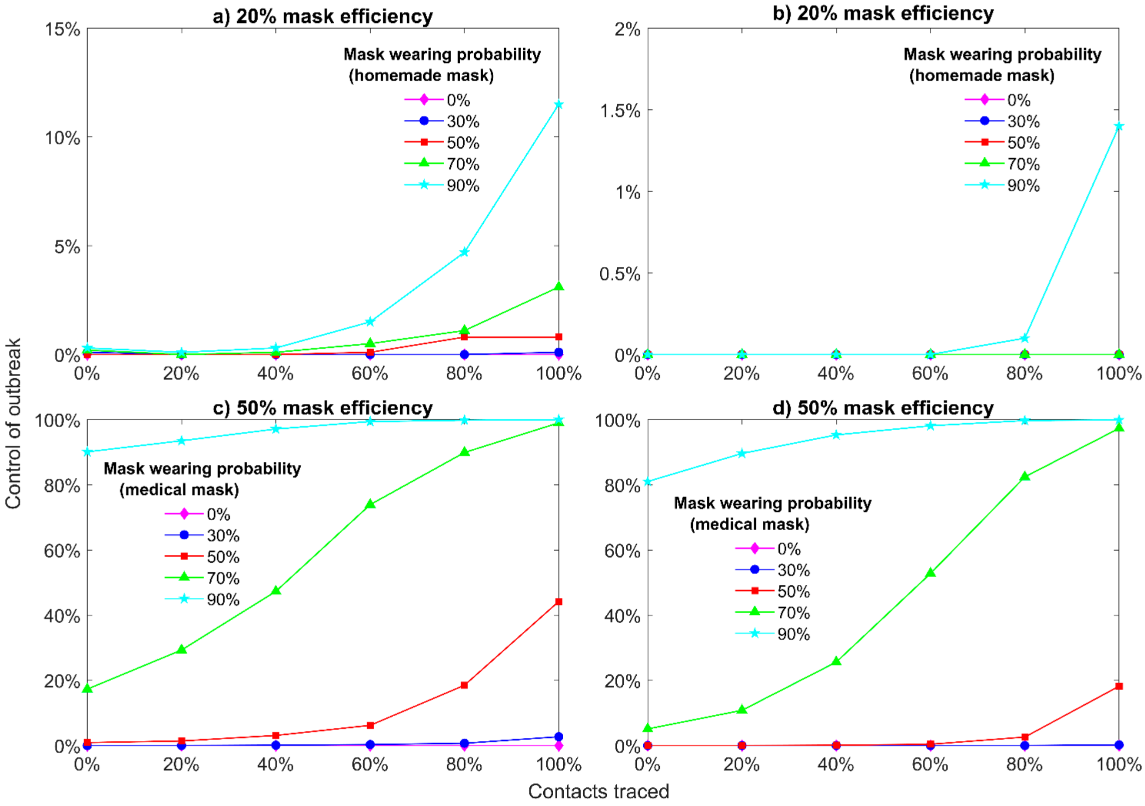

Supplementary Figure S3. Outbreak control for different number of initial symptomatic (sym.) and asymptomatic (asym.) cases. $R_0 = 2.5$, $R_a = 1.875$, 4 days of mean delay, 40% transmission before symptoms, all people wear masks and the mask hinders spreading and protects from getting infection with different mask efficiency e_m: **a)** $e_m = 20\%$ (homemade mask), sym. = 50, asym. = 50; **b)** $e_m = 20\%$ (homemade mask), sym. = 100, asym. = 100; **c)** $e_m = 50\%$ (medical mask), sym. = 50, asym. = 50; **d)** $e_m = 50\%$ (medical mask), sym. = 100, asym. = 100.

4. COVID-19 wave in Taiwan during May-June 2021

Official data of the new COVID-19 wave in Taiwan that was started in May 2021 [7]. From the dynamics of new cases (Figure S4) we can mention that there is no witnessing an expected exponential growth in cases. This further proves that the control measures (mandatory medical mask wearing everywhere and high proportion of traced contacts) effectively kept the basic reproduction number reasonably around the value of one.

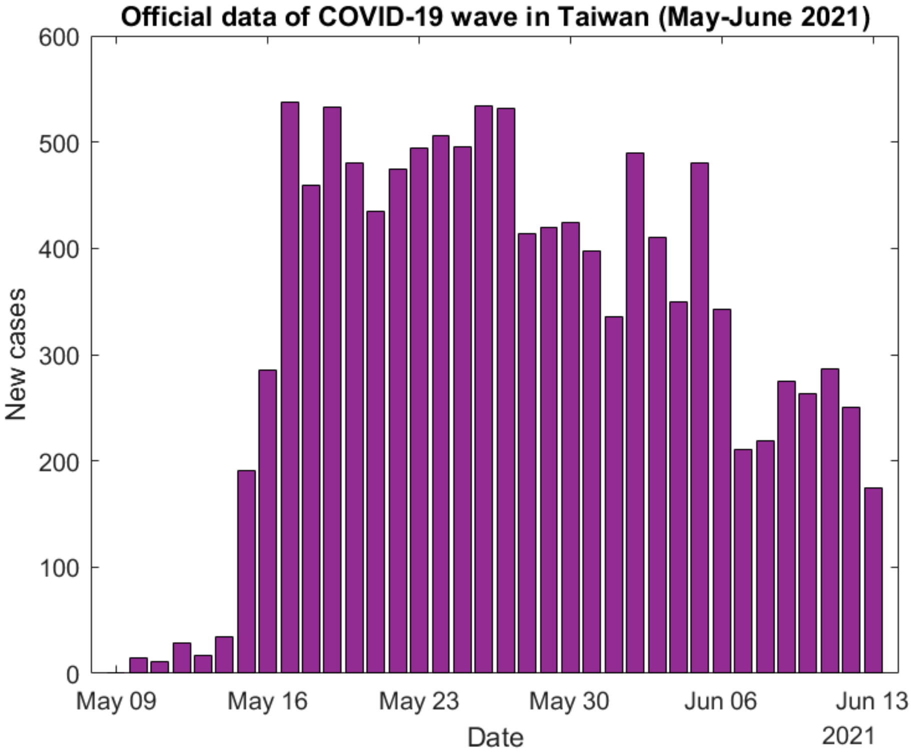

Supplementary Figure S4. Official new cases in Taiwan during May-June 2021.

References

1. Endo, A.; Centre for the Mathematical Modelling of Infectious Diseases COVID-19 Working Group; Abbott, S.; Kucharski, A.J.; Funk, S. Estimating the overdispersion in COVID-19 transmission using outbreak sizes outside China [version 3; peer review: 2 approved]. *Wellcome Open Res.* **2020**, *5*, 67 (https://wellcomeopenresearch.org/articles/5-67/v3).
2. Johnson, N.L.; Kemp, A.W.; Kotz, S. Univariate discrete distributions. 3rd ed.; Wiley: Hoboken, New Jersey, USA, 2005.
3. Wong, F.; Collins, J. J. Evidence that coronavirus superspreading is fat-tailed. *Proc. Natl. Acad. Sci. USA* **2020**, *117*, 29416-29418.
4. Donnelly, C.A.; et al. Epidemiological determinants of spread of causal agent of severe acute respiratory syndrome in Hong Kong. *Lancet* **2003**, *361*, 1761-1766.
5. Backer, J.A.; Klinkenberg, D.; Wallinga, J. Incubation period of 2019 novel coronavirus (2019-nCoV) infections among travellers from Wuhan, China. *Euro Surveill.* **2020**, *25*, 2000062.
6. Hellewell, J.; et al. Feasibility of controlling COVID-19 outbreaks by isolation of cases and contacts. *Lancet Glob. Health.* **2020**, *8*, e488-e496.
7. Taiwan Centers for Disease Control. Press Releases. Available online: https://www.cdc.gov.tw/En/Bulletin/List/7tUXjTBf6paRvrhEl-mrPg (accessed on 13 June 2021).
